# Pharmacological and Biological Efficacy of Chitosan-Based Materials

**DOI:** 10.3390/ijms262110735

**Published:** 2025-11-04

**Authors:** Anathi Dambuza, Pennie P. Mokolokolo, Mamookho E. Makhatha, Motshabi A. Sibeko

**Affiliations:** 1 Department of Chemistry, University of the Free State (QwaQwa Campus), Kestell Road, QwaQwa, Phuthaditjhaba 9866, South Africa; mokolokolopp@ufs.ac.za (P.P.M.); sibekoma@ufs.ac.za (M.A.S.); 2 Department of Metallurgy, University of Johannesburg, Doornfontein Campus, 55 Beit St, Doornfontein, Johannesburg 2028, South Africa; emakhatha@uj.ac.za

**Keywords:** chitosan, non-communicable diseases, clinical trial, wound healing, biocompatible polymer, pharmacological applications

## Abstract

Chitosan (CS) is a biodegradable and biocompatible polysaccharide, obtained by the deacetylation of chitin. It has gained significant attention as a versatile material for biomedical applications due to its mucoadhesive properties, ease of chemical modification and intrinsic pharmacological activities. This review synthesizes two decades (2005–2025) of literature, focusing on chemical modifications of chitosan for pharmacological purposes and their therapeutic implications in non-communicable diseases (NCDs) and wound healing. Evidence highlights the roles of chitosan-based materials in anticancer, anti-inflammatory, antidiabetic, antihypertensive, and neuroprotective activities, alongside their integration in advanced wound healing strategies. Clinical trials have demonstrated the translational potential of chitosan-based materials. In general, chitosan-based materials exhibit promising dual functions as bioactive agents and drug carriers, necessitating additional investigation in clinical and regulatory frameworks to accelerate therapeutic adoption. In contrast to other studies, this study offers a mechanistic and integrative viewpoint that links chitosan’s chemical modification techniques with their pharmacological effects and clinical translation potential, providing novel perspectives on structure–activity correlations and therapeutic design.

## 1. Introduction

Non-communicable diseases (NCDs) are classified as a group of chronic illnesses that cannot be transmitted from one person to another. These encompass a range of conditions including cancer, diabetes, neurodegenerative disorders and chronic inflammatory disease, and are among the leading causes of morbidity and mortality worldwide. It is estimated that around 70% of deaths are NCD-related [1]. NCDs have placed a deep burden on health care facilities and systems, notably in low and middle-income countries; for instance, 80% of NCD-related deaths occur in these countries. Key risk factors include unhealthy diets (particularly those high in saturated fats), tobacco use, and physical inactivity [2,3]. Conventional pharmacological treatments for these conditions are often limited by poor bioavailability, rapid metabolism, and inadequate tissue targeting [4,5]. These shortcomings highlight the urgent need for improved drug delivery strategies and safer therapeutic agents.

**Figure 1:**
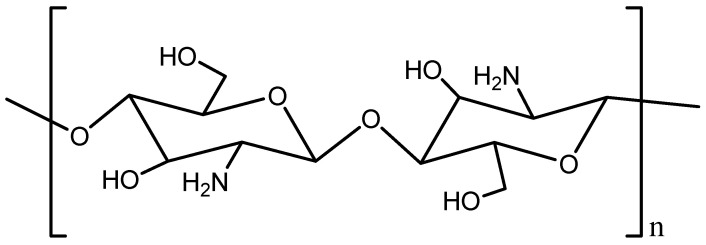
Chemical structure of chitosan.

Natural products are receiving growing attention in drug discovery, and chitosan-based materials are among the most promising candidates. Chitosan is a biocompatible, biodegradable, and low-toxicity polymer obtained through the deacetylation of chitin. Structurally, it is a versatile linear biopolymer composed of β-(1→4)-linked 2-amino-2-deoxy-D-glucose (D-glucosamine) units (see Figure 1). Its physicochemical properties, including a positive surface charge [6], mucoadhesiveness [7], and ease of chemical modification [8], make it highly suitable for diverse pharmacological applications. Beyond its role as a drug carrier, chitosan also exhibits intrinsic pharmacological activities, including anticancer, anti-inflammatory, antimicrobial, antioxidant, and antidiabetic effects [9,10,11,12,13]. However, the mechanistic and pharmacodynamic contributions remain only partially understood.

**Figure 2:**
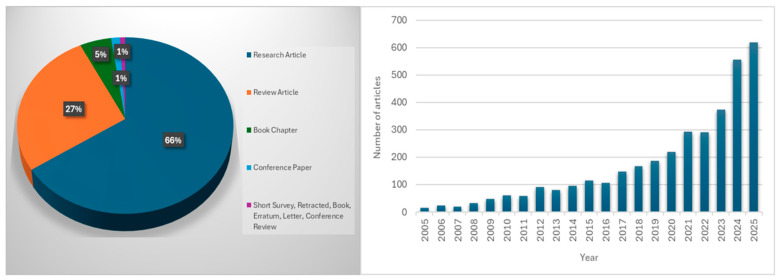
Number of published articles on “therapeutic AND properties AND of AND chitosan AND chitosan” based on the Scopus database (accessed on 15 September 2025).

Scopus database analysis (2005–2025, English-language articles) indicates growing research interest in chitosan for therapeutic applications, with 3598 publications currently available (Figure 2). Numerous review articles have focused on the general uses of chitosan derivatives in biomedical and pharmaceutical contexts. These include studies on the design of encapsulated polyphenolic compounds, antimicrobial activities of chitosan, the role of biopolymers in drug delivery, and the use of chitosan biopolymer and its modified nanocomposites in pharmaceuticals. Other reviews have also highlighted the therapeutic potential of chitosan and its derivatives in the treatment of osteoarthritis as well as a wide variety of bioactivity supporting its versatile role as a bio-macromolecule in biomedical applications [14,15,16,17,18,19]. Few review articles have provided a comprehensive and mechanistic overview linking chemical modifications of chitosan to their pharmacological activities across the spectrum of NCDs, including wound healing. Therefore, this review provides insights into the novel approach and offers an integrated perspective on the pharmacological applications and mechanistic interactions of chitosan-based materials, including their use in the treatment of NCDs and wounds.

## 2. Methodology

This article was prepared as a traditional narrative review article; nevertheless, data collection in this study was conducted using multiple databases to ensure the information was comprehensive, aligned, and dynamic. Reputable online databases such as Google Scholar, Scopus, PubMed, and Web of Science were utilized to facilitate this process. The search targeted reliable and high-quality studies from 2005 to 2025 (two decades), aiming for the most recent studies. Data collection involved a thorough review of peer-reviewed journals, scientific articles, review articles, and book chapters in pharmacology, toxicology and therapeutics, chemistry, biochemistry, immunology, material sciences, and related fields. The keywords used included “Pharmacological Applications of Chitosan AND Its Derivatives,” “Biological potency of chitosan AND its derivatives,” “Modifications of chitosan for pharmacological applications,” and “Chitosan Derivatives AND Their Biological Efficacy”. Selected articles were categorized based on modifications of chitosan for therapeutic or pharmacological applications and their roles in pharmacological or biological efficacy (e.g., anti-inflammatory, anticancer, neuroprotective, metabolic disorders, wound healing). The synthesized results were integrated into a cohesive discussion, and the overall therapeutic efficacy of chitosan-based materials across various biological models. Only articles written in English were included.

## 3. Chemical Modifications and Functionalization Strategies

**Figure 3:**
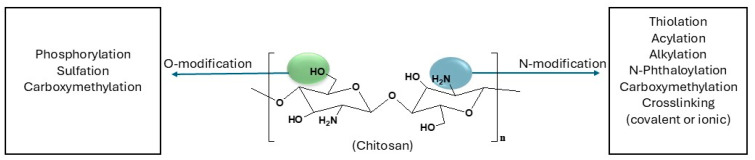
Chemical modifications of chitosan for biomedical applications [23,24].

Chitosan is a naturally occurring polysaccharide derived from the deacetylation of chitin and is mostly extracted from the shells of crustaceans, insects, and fungi [20]. It has gained extensive interest in the biomedical field due to its inherent biocompatibility, biodegradability, and bioactivity [21]. However, its pharmacological applications are limited by poor solubility at physiological pH and variability in physicochemical properties [22]. Some of the modifications made to chitosan for biomedical applications are shown in Figure 3, along with the functional groups that are most favored by these modifications.

### 3.1. Acylated CS

**Figure 4:**
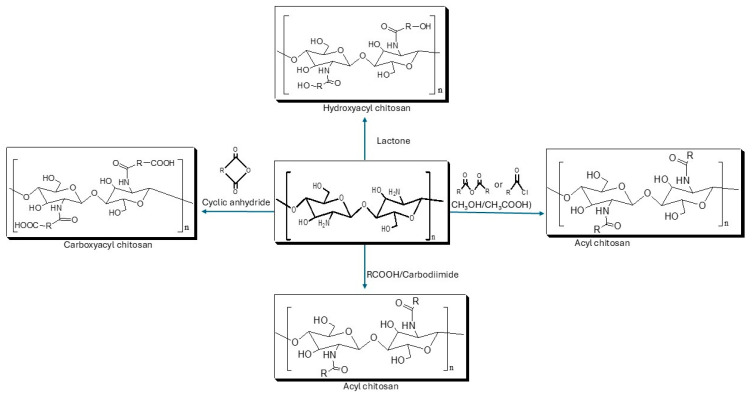
Acylation of chitosan.

Acylation of chitosan involves the introduction of acyl groups in the chitosan chain, on either the amino or hydroxyl group, though mostly on the amino group. It is one of the most frequently used modifications of chitosan, which involves reacting chitosan with various organic acids and their derivatives, such as anhydrides and acyl chlorides, to incorporate aliphatic or aromatic acyl groups into the chitosan chain (see Figure 4) [25]. Acylation improves the physicochemical and biomedical properties of chitosan, particularly solubility, stability, and hydrophobic interactions [26,27,28]. For example, Almeida et al. [29] synthesized an amphiphilic O-acylated chitosan derivative that self-assembled into micelles. This derivative self-assembles into micelles capable of encapsulating a hydrophobic anticancer drug (camptothecin) with high efficiency (78%), providing controlled release and stabilization of the active drug form.

**Figure 5:**
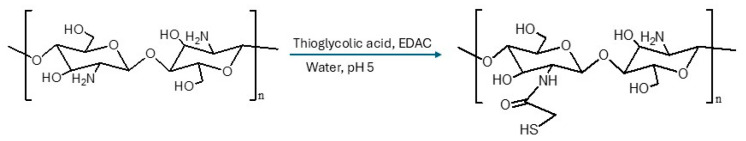
Synthesis of Thiolated Chitosan [34].

A special subclass of acylation reactions involves the use of thiol-containing carboxylic acids, such as thioglycolic acid, to introduce sulfhydryl (–SH) functional groups through acylation. Thiolation of chitosan was made based on the hypothesis that a disulfide bond will be formed between the polymer-bound free thiol groups and the thiol group present in mucus [30]. See its synthetic protocol in Figure 5. It has been reported that thiolated chitosan improves the mucoadhesive strength and permeability [31,32]. Additionally, the creation of intramolecular and intermolecular disulfide bonds results in a compact, three-dimensional network with increased cohesiveness, enabling regulated drug release [33].

### 3.2. Alkylated Chitosan

**Figure 6:**
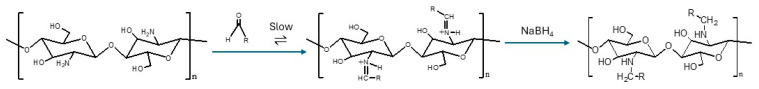
Reductive alkylation of chitosan [38].

The introduction of an alkyl group on the chitosan chain significantly weakens intermolecular hydrogen bonds, thereby improving solubility for biomedical applications [35]. The hydrophobic nature of alkyl groups means that solubility can be tuned by varying chain length: short chains enhance solubility, while long chains reduce it [36,37]. Alkylated chitosan derivatives can be prepared through the reductive alkylation of chitosan. Reductive alkylation of chitosan proceeds through imine formation between chitosan amine groups and aldehydes, followed by NaBH_4_ reduction to produce N-alkyl chitosan derivatives (see Figure 6) [38]. Alkylated chitosan has been used extensively in the treatment of wounds and hemostasis therapy [39,40,41,42,43].

### 3.3. N-Phthaloylation of Chitosan

N-phthaloylation of chitosan is another method of modifying chitosan that is mostly used for biomedical applications. This modification introduces phthalimide groups into the chitosan chain. These groups act as a protective group which further increases the solubility and stability of the chitosan, enabling selective click chemistry or polymer grafting which results in functional derivatives such as micellar systems, tailored scaffolds, and stimuli-responsive drug carriers [44].

### 3.4. Other Chitosan Modifications

Other types of modifications and their applications are discussed in Table 1. It is important to note that the optimal modification depends on the intended application; for instance, overall, among the various chitosan-based materials listed in Table 1 below, carboxymethyl chitosan stands out as a broadly applicable material for wound dressing, owing to its high-water solubility, excellent biocompatibility, and intrinsic antimicrobial activity, while phosphorylated chitosan is preferred for bone tissue engineering due to its calcium-binding and osteoconductive properties [45,46]. For drug delivery systems, thiolated chitosan is most advantageous owing to its mucoadhesive and permeation-enhancing effects [31].

## 4. Chitosan-Based Materials’ Efficacy in Wound Dressing and Healing

**Figure 7:**
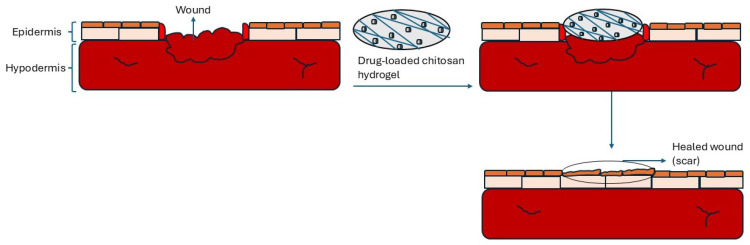
Chitosan-based material in wound dressing.

Chitosan-based materials have been reported to play a major role in wound dressing since they promote cell growth, minimize scarring, have good clotting capabilities, and absorb exudate (see Figure 7) [69]. For instance, Zhang et al. [70] synthesized a near-infrared (NIR)-responsive double-network hydrogel, composed of chitosan, which was incorporated with hollow copper sulfide nanoparticles (HCuS) and sodium nitroprusside (SNP). The hydrogel showed excellent mechanical stability, strong antibacterial activity (>99% MRSA elimination), anti-inflammatory effects, and accelerated wound healing (93% closure in 9 days). Kumi et al. [71] designed a 3D printed chitosan-based porous flexible hydrogel electrode, creating a material that conforms to wounds for electrical stimulation treatment. The chitosan-based material demonstrated a tensile strength of around 2.43 MPa, stretchability of approximately 48.9%, and a hydrophilic, porous structure that improved biocompatibility and moisture retention. In vivo diabetic wound models demonstrated faster closure (~99% by Day 14) with improved re-epithelialization and collagen deposition, highlighting its potential for customized wound healing applications. It also achieved >85% bacterial suppression against *Methicillin-resistant Staphylococcus aureus* (MRSA) and *Escherichia coli* (*E. coli*).

In vivo study conducted by Xiao et al. [72] on mice showed that acid-responsive hydrogels based on carboxymethyl chitosan can efficiently accelerate wound healing. According to bacterial culture data, the chitosan hydrogel had good antibacterial action. Lastly, there was a remarkable increase in the production of M1 macrophages for pathogen clearance and a switch to M2 macrophages to assist tissue regeneration, demonstrating the hydrogel’s capacity to alter the Reactive Oxygen Species (ROS)-macrophage axis. Ren et al. [73] prepared a chitosan/glycerophosphate (CS/GP) hydrogel loaded with hydrothermally prepared Cu_3_SnS_4_ nanoparticles to construct a novel wound dressing (CS/GP/Cu_3_SnS_4_). The hydrogel showed >95% antibacterial activity against *Staphylococcus aureus* (*S. aureus*) and *E. coli*, with confirmed biocompatibility in vitro. In vivo studies demonstrated superior burn wound healing. A chitosan-based hydrogel (COG-Z@P200) containing polydopamine-coated ZIF-8 nanoparticles was synthesized in the study by Gao et al. [74] to work in tandem with metabolic-immune reprogramming and moderate photothermal treatment. The chitosan-based hydrogel provided an antibiotic-free strategy against resistant infections by achieving >99.5% eradication of MRSA, reprogramming macrophages towards pro-healing phenotypes, enhancing angiogenesis, and accelerating diabetic wound repair by 48%.

Yu et al. [75] synthesized a CS/ZnO-NPs@Exos hydrogel by combining zinc oxide nanoparticles, exosomes, and chitosan, which produced prolonged exosome release in diabetic wounds. The hydrogel showed great therapeutic promise for the healing of diabetic skin injuries by speeding wound closure, lowering inflammation, facilitating re-epithelialization, collagen deposition, and angiogenesis. Methacrylate-grafted quaternary ammonium chitosan, polyvinyl alcohol, ZIF-8, and nicotinamide mononucleotide were used by Zhang et al. [76] to create a multifunctional hydrogel that could be applied to irregular wounds. With the help of quaternary ammonium groups in chitosan and Zn^2+^ release, the hydrogel exhibited long-lasting antibacterial action, which in turn led to decreased infection, improved epithelium regeneration, and collagen deposition in vivo. Extensive research by Salarizadeh et al. [77] created a two-layer sponge wound dressing by combining polyvinyl alcohol, sodium alginate, and a chitosan active layer that was freeze-dried and contained zinc oxide nanoparticles and *Hyssopus officinalis* extract. The dressing’s antimicrobial activity, clotting ability, and porosity were all good. After 14 days, in vivo burn models showed superior biocompatibility and quicker healing, with 92.7% wound closure as opposed to 73.2% with Alpha ointment.

A multifunctional hemostatic bandage has also been created by anchoring silica nanoparticles onto polydopamine-coated chitosan non-woven fabric, combined with polyphosphate. The bandage showed strong flexibility, cytocompatibility, adhesion, and antibacterial activity, while promoting coagulation and hemostasis. In rat liver and femoral artery injury models, PSPC achieved hemostatic performance comparable to QuickClot Combat Gauze^®^ and significantly enhanced full-thickness wound healing [78]. Self-healing hydrogel was created by Zhou et al. [79] using guanidinium-functionalized chitosan (GCS) and aldehyde-modified chitosan (ACS) and a Schiff base reaction. The hydrogel exhibited potent antibacterial and anti-inflammatory properties. It outperformed commercial Aquacel™ Ag^+^ dressings in infected full-thickness rat wounds, achieving 94.5% closure in 14 days after infection. Dai et al. [80] created a cellulose-based composite hydrogel by mixing acrylamide, carboxymethyl chitosan, and cellulose dialdehyde to create a double network using free radical polymerization and the Schiff base reaction. High levels of toughness, stretchability, tensile strength, self-adhesion, and biocompatibility were all demonstrated by the chitosan-based hydrogel. The full-thickness wound model in mice showed a considerable acceleration in healing.

Another research developed a chitosan-based Janus wound dressing with asymmetric wettability, consisting of a methacrylated chitosan (CSMA), incorporating polydopamine-coated silica (PDA-SiO_2_) hydrophilic sponge for hemostasis and angiogenesis, and a TPU/ZIF-8 nanofiber outer layer for antibacterial protection. The dressing showed strong bacterial resistance, promoted coagulation, and in vivo reduced bleeding time and blood loss while enhancing collagen deposition, angiogenesis, and full-thickness wound healing [81]. Purified chitosan carbonate liquid dressing via an ammonium bicarbonate-mediated acetate replacement strategy was also synthesized. In full-thickness wound models, it promoted rapid repair with hair follicle regeneration within 10 days, outperforming commercial benchmarks like 3M Cavilon [82]. Another injectable multifunctional hydrogel (RM-CHβ) using chitosan hydrochloride, hydroxyethyl cellulose, β-glycerol phosphate, and resveratrol micelles was used to treat *S. aureus*-infected full-thickness wounds. The hydrogel showed strong adhesion, antioxidant, antibacterial, and hemostatic properties, while in vivo it accelerated healing by enhancing angiogenesis, collagen deposition, and inflammation control [83]. A wide range of chitosan-based materials have shown great promise as wound dressings due to their distinct physicochemical characteristics, which include superior adhesion, swelling capacity, biodegradability, and increased mechanical strength. Furthermore, a lot of these systems include inherent or functionalized hemostatic, antioxidant, and antibacterial properties, which support their function in hastening tissue regeneration and wound healing [84,85,86,87,88]. Table 2 highlights some of the recent studies (strictly 2024–2025) on chitosan-based materials in wound healing. In these studies, rats, mice, and rabbits are used because they provide well-characterized, reproducible in vivo models with healing properties closer to human skin [89,90].

## 5. Biological Activities and Clinical Trials

Beyond wound healing, chitosan-based materials exhibit a wide range of biological activities, which further underscore their pharmacological versatility. These biological activities include anticancer, anti-inflammatory, antidiabetic, antihypertensive, and neuroprotective effects, many of which share underlying mechanisms, such as modulation of oxidative stress, control of inflammation, and enhanced tissue regeneration, previously discussed in the context of wound repair.

### 5.1. Anticancer Activity

Cancer is a chronic illness that is mostly caused by the body’s cells proliferating out of control, producing tumors that may or may not be malignant [104]. Numerous anticancer medications on the market today have unfavorable pharmacological characteristics, such as low aqueous solubility, irritation, instability, fast metabolism, and nonselective drug distribution. These characteristics can lead to negative outcomes, such as suboptimal therapeutic activity, dose-limiting side effects, and poor patient quality of life [105,106,107,108,109,110]. Hence, scientists are looking for less harmful and effective medications for cancer victims. One naturally occurring polysaccharide, considered to have anticancer properties, is a chitosan-based material. For instance, the most recent research by Yu et al. [111] created a gellan gum/chitosan-based bilayer scaffold, loaded with green tea extract and curcumin, and it demonstrated potent antibacterial and antioxidant properties. When tested on MCF-7 breast cancer cells, the scaffold remained biocompatible with normal fibroblasts but decreased cell viability from around 92% (control) to about 23%. Another work related to anticancer properties (breast cancer) of chitosan-based materials was demonstrated by Mirzaie et al. [112] where they developed Docetaxel–chitosan nanoparticles (DTX–CS NPs), assembled with hyaluronic acid, and tested them against MCF-7 breast cancer cells. The nanoparticles showed enhanced cellular uptake, apoptosis induction, and altered gene expression, with a significantly higher BAX/BCL-2 ratio compared to free Docetaxel. Importantly, the material exhibited lower cytotoxicity toward normal fibroblasts, suggesting improved selectivity and reduced off-target toxicity. Mirzaie et al. [112] examined the effects of chitosan nanoparticles loaded with ethanolic and n-hexane extracts from Moringa oleifera seeds on MCF-7 breast cancer cells. The IC_50_ values for both nano formulations were 1843 µg/mL and 382 µg/mL, respectively, indicating dose-dependent cytotoxicity. They dramatically decreased the expression of genes linked to metastasis (Wnt/β-catenin, cyclin D1, TGF-β, and Snail) and inhibited cell proliferation (Ki-67 marker). Chitosan-coated PLGA nanoparticles loaded with ursolic acid for breast cancer therapy were tested on MCF-7 and MDA-MB-231 breast cancer cells. The chitosan-based nanoparticles showed high encapsulation efficiency of around 80%, controlled release, and significant cytotoxicity compared to free ursolic acid [113].

Poly(ethylene glycol) methacrylate-grafted chitosan (PEGMA-g-Cs) microbeads with gold-coated SPIONs were created by Olaoye et al. [114] for the transport of lactoferrin to the colon. The formulation demonstrated decreased cancer cell survival, pH-responsive release, and good encapsulation efficiency when tested against Caco-2 colorectal cancer cells. In studies on HCT-116 colon cancer cells, the chitosan–graphene oxide–silver (Cs/GO/AgNP) nanocomposite demonstrated dose-dependent cytotoxicity with around 72% reduction in cell viability at 100 µg/mL, showing high anticancer activity [115]. Chang et al. [116] demonstrated that colon cancer cells (HT29, DLD-1, HCT116, SW480) cultured on chitosan membranes acquired stronger stem-like features, enhanced motility, drug resistance, self-renewal, and upregulation of stemness markers by activation of the canonical Wnt/β-catenin-CD44 signaling pathway.

Qu et al. [117] created anisamide-functionalized, pH-responsive chitosan micelles for paclitaxel delivery, which demonstrated improved uptake in sigma-1 receptor–positive prostate cancer cells (PC-3), pH-triggered drug release, stronger tumor inhibition in vivo, and decreased systemic toxicity. Another study that demonstrated that chitosan-based materials have anticancer properties (prostate cancer) was performed by Khan et al. [118]. The study showed that oral chitosan encapsulated with epigallocatechin-3-gallate (EGCG) nanoparticles enhances bioavailability, inhibits prostate tumor growth, reduces PSA, induces apoptosis, and suppresses proliferation and angiogenesis, offering a promising nanochemopreventive strategy for prostate cancer. The effectiveness of chitosan-based materials has also been confirmed in /DU145, LNCaP, and PC-3 cell models, demonstrating strong anticancer potential in prostate cancer by suppressing cell proliferation, significantly reducing tumor size, and persistently inhibiting tumor growth [119,120,121].

### 5.2. Anti-Inflammatory Activity

Inflammation is the second body defense mechanism. In reaction to viruses, irritants, and damaged cells, among other damaging and alien stimuli, the immune system uses this mechanism to identify, reject, and start the healing process. Chronic inflammation can persist for months or years. The source of the damage and the body’s capacity to heal and reverse it usually dictate the degree and length of chronic inflammation [122,123]. Numerous studies have been carried out to investigate the potential of chitosan-based formulations in inflammation. Xu et al. [124] reviewed that chitosan and its derivatives show anti-inflammatory activity mainly through cytokine modulation and signaling pathway regulation. For instance, Zhong et al. [125] found that the deferoxamine-loaded chitosan–chlorogenic acid/oxidized hyaluronic acid hydrogel (CCOD) dramatically reduces inflammatory responses in diabetic and infected wound models by upregulating the anti-inflammatory cytokine IL-10 and downregulating pro-inflammatory cytokines (IL-1β, IL-6, TNF-α, and CRP). Goyal et al. [126] found that chitosan-based nanocarriers primarily induce anti-inflammatory effects by modulating cytokines, suppressing MCP-1–mediated monocyte infiltration, and downregulating TNF-α, IL-1β, and IL-6, while simultaneously promoting tissue repair. This was confirmed by the ability of the material to reduce joint inflammation, synovial hyperplasia, and intestinal barrier defects in models of rheumatoid arthritis and inflammatory bowel disease.

Lu et al. [127] created a gallic acid–chitosan methacrylate (GA-CS-MA) hydrogel that effectively suppressed pro-inflammatory signals, reduced ROS and Nitric Oxide (NO) production, and modulated macrophage polarization in response to Lipopolysaccharide (LPS) stimulation. Its potent anti-inflammatory properties were validated through in vitro and in vivo studies, revealing decreased cell infiltration, thinner fibrous capsules, and increased M2 polarization. Chitosan nanoparticles have also been proven to inhibit the inflammation response of LPS-stimulated macrophages, by altering common inflammation variables and biomarkers [128]. Guan et al. [129] demonstrated that Ibuprofen-chitosan methacrylate (IBU-CS-MA) hydrogel dramatically decreases the amount of ROS that LPS-stimulated macrophages produce. It is well recognized that ROS contributes to inflammation and oxidative damage. Numerous studies have demonstrated that chitosan-based materials reduce inflammation through various mechanisms by suppressing NF-κB, which downregulates pro-inflammatory mediators (IL-1β, IL-6, IL-12, and TNF-α) and decreases the production of NO, IL-6, and TNF-α in macrophages. Additionally, they stimulate inflammation resolution and tissue repair by promoting angiogenesis and enhancing anti-inflammatory cytokines (TGF-β, IL-10) [130,131,132,133,134].

### 5.3. Antidiabetic Activity

Diabetes is a general term for several metabolic diseases caused by partial or total insulin insufficiency and marked by hyperglycemia. It is a long-term condition that impacts how your body uses food as fuel. The body transforms most of the food we eat into glucose, or sugar, which is then delivered into the bloodstream [135,136]. Chitosan-based formulations have shown potential as a key remedy to treat diabetes. The potential of chitosan to address the shortcomings of traditional antidiabetic treatments by enhancing medication bioavailability, lowering insulin resistance, and improving glycaemic control makes it practically significant [137,138]. For instance, Wadasinghe et al. [139] demonstrated that chitosan–tripolyphosphate nanoparticles (CS–TPP) encapsulating *Gmelina arborea* and *Spondias pinnata* extracts (GAE–CS–TPP, SAE–CS–TPP) exhibited strong antidiabetic potential, with high encapsulation efficiency and enhanced inhibitory activity against α-glucosidase and DPP-IV enzymes compared to crude extracts, thereby supporting their role in controlling hyperglycemia in diabetes. Abdel-Baky et al. [140] conducted another in vitro study which also demonstrated strong anti-diabetic effects. They found that chitosan-quinoline Schiff base increased glucose uptake and achieved high inhibition of key enzymes that break down carbohydrates (92.10% for α-glucosidase and 99.78% for α-amylase). These findings were further supported by molecular-docking analysis and a favorable safety profile.

Many in vivo studies have also been carried out to evaluate the antidiabetic activities of chitosan-based formulations. According to research by Priyanka et al. [138] male Wistar rats with type 1 diabetes (T1DM) show significant anti-hyperglycemic benefits from low-molecular-weight chitosan (LMWC), a chitosan derivative, mostly by modulating the AKT/PI3K/FOXO pathway to enhance insulin sensitivity and glucose metabolism. Yang et al. [141] also performed the in vivo study, which demonstrated that a chitosan-based hydrogel combined with umbilical cord mesenchymal stem cells (UC-MSCs) can significantly enhance glucose regulation and restore pancreatic islet integrity in Type 2 Diabetes Mellitus (T2DM) mice by stimulating β-cell function and promoting macrophage polarization toward the M2 phenotype. Furthermore, chitosan-based embelin nanoparticles (ECNPs) significantly lowered blood glucose, as demonstrated by Maanvizhi et al. [142] in streptozotocin-induced diabetic rats, showing dose-dependent antidiabetic activity comparable to glibenclamide and demonstrating good histological safety up to 25 mg/kg, highlighting their potential for hyperglycemia management. Similarly, chitosan extracted from crayfish shells through acid demineralization and alkali deproteinization showed marked anti-hyperglycemic activity in alloxan-induced diabetic rats, with the chitosan-only group returning blood glucose to near-normal levels within 2 h postprandially, highlighting chitosan’s ability to improve glucose tolerance and enhance the efficacy of oral hypoglycemic agents [143].

### 5.4. Anti-Hypertensive Activity

Hypertension (high blood pressure) is a chronic disorder in which the blood pressure in the arteries is too high. Hypertension is a significant public health problem in both developing and developed countries [144]. Research on the antihypertensive potential of chitosan-based materials has gained importance, driven by the worldwide prevalence of hypertension and its contribution to cardiovascular diseases, responsible for over 17.5 million deaths each year and expected to rise by >23.6 million by 2030 [145,146]. As an example, Chitosan/carboxymethyl-cellulose (CS/CMC) biomaterials containing captopril were used by Kim et al. [147] as a chitosan-based antihypertensive system by releasing the drug by non-Fickian diffusion through artificial skin and pseudo-Fickian diffusion in buffer. They demonstrated excellent angiotensin-converting enzyme (ACE)-blocking action for blood pressure regulation in an artificial skin model, achieving sustained release for 36 h and inhibiting ACE by up to 88.4%. Batista et al. [148] conducted research that resulted in the development of chitosan microparticles-in-films that were loaded with the antihypertensive peptide KGYGGVSLPEW. These microparticles were able to reduce blood pressure by inhibiting ACE and enhancing buccal absorption. The efficacy of these microparticles was demonstrated in vitro using the human buccal epithelium TR146 cell model, demonstrating rapid film disintegration and controlled peptide release. In a related study, Auwal et al. [149] developed chitosan–antihypertensive biopeptide nanoparticles (Chit-AntBiop-NPs) that lower blood pressure by inhibiting ACE and providing sustained peptide release. Their efficacy was evaluated in vivo using hypertensive rats, achieving a dose-dependent systolic blood pressure reduction of up to 59.8 ± 7.7 mmHg at 6 h post-administration. Du et al. [150] also reported that water-soluble chitosan (WSC) lowers blood pressure by inhibiting vascular remodeling through suppression of Nuclear Factor of Activated T-Cells, cytoplasmic 1 (NFATc1) expression and c-myc–mediated smooth-muscle proliferation, as shown in vitro in primary rat aortic smooth-muscle cells and in vivo in spontaneously hypertensive Wistar-Kyoto rats treated with 150 mg/kg/day WSC.

Another study by Khalid Danish et al. [151] used spontaneous hypertensive rats (SHR) to confirm that chitosan–zein nanoparticles (CZ NP) encapsulating the antihypertensive tripeptides IPP and LKP were effective through sustained release and ACE inhibition, which enhanced peptide stability and bioavailability. Oral administration of the nanoparticles lowered systolic blood pressure for up to 8 h. Extending these findings, the antihypertensive effects of chitosan-based materials, such as chitosan–ferulic acid salts, miR-29b–chitosan nanoparticles, and chitooligosaccharide–polyphenol conjugates, are mediated by distinct mechanisms, including bile acid adsorption and ferulic acid release, enhanced nitric oxide bioavailability with antifibrotic action, and ACE/renin mixed-type inhibition. Their effectiveness was confirmed in angiotensin II- or SHR, showing notable reductions in blood pressure and associated complications [152,153,154].

### 5.5. Neuroprotective Activity

Neurological illnesses impact the brain, spinal cord, and the peripheral nervous system. Anxiety, depression, stroke, Alzheimer’s, Parkinson’s, and other similar illnesses are among them. The growing incidence of neurodegenerative diseases and neurological injuries, which pose serious global health challenges, has made the investigation of chitosan-based materials for their neuroprotective potential a critical research area [155,156]. Recent research indicates that chitosan is a good candidate in neurotherapeutics since it promotes neural repair, enhances drug bioavailability, and modulates neuroinflammation [157,158]. For instance, according to the research conducted by Dadkhah et al. [159] Fluoxetine-loaded pegylated chitosan nanoparticles significantly enhanced the neuroprotective effects in a rat model of hippocampal demyelination, improving memory function, reducing anxiety-like behaviors, increasing Brain-Derived Neurotrophic Factor (BDNF) levels in the hippocampus, and more effectively reducing demyelination lesions compared to fluoxetine alone. Similarly, in a rat model of intracerebral hemorrhage (ICH) stroke, Lin et al. [160] created a chitosan micellar self-healing hydrogel (CMD hydrogel), which showed in vivo neuroprotective efficacy. Significant brain tissue regeneration and functional improvement were demonstrated by approximately 84% behavioral recovery, balanced brain midline shift, increased neurogenesis (doublecortin/nestin-positive cells), and promoted angiogenesis that resulted from intracerebral injection of CMD hydrogel.

It is worth noting that chitosan is compatible with other materials, as Wang et al. [161] demonstrated that valproic acid–labeled chitosan nanoparticles (VA-CN) exhibited strong in vivo neuroprotective effects in a rat spinal cord injury (SCI) model. VA-CN treatment significantly improved tissue repair and locomotor function, enhanced neural stem cell proliferation and differentiation (Nestin^+^/Ki67^+^ and Tuj-1^+^ cells), increased neurotrophic factors (BDNF, NGF, NTF-3), and reduced microglial activation compared with chitosan or valproic acid alone. Saleem et al. [162] found that chrysin-loaded chitosan nanoparticles were neuroprotective in an Aβ1-42-induced Alzheimer’s zebrafish model, lowering neuronal apoptosis, oxidative stress, and amyloid aggregation while maintaining synaptic integrity. These nanoparticles also boosted learning and memory ability in many behavioral tests. Overall, Chitosan-based materials, such as 3D scaffolds, dopamine-loaded nanoparticles, magnoflorine-collagen nanocapsules, and citric acid cross-linked hydrogels, demonstrated in vitro neuroprotective effects by supporting neural cell survival, enhancing antioxidant enzyme activity, reducing oxidative stress, and inhibiting acetylcholinesterase, highlighting their potential for nerve repair and treatment of neurodegenerative diseases [163,164,165,166].

### 5.6. Clinical Studies

**Figure 8:**
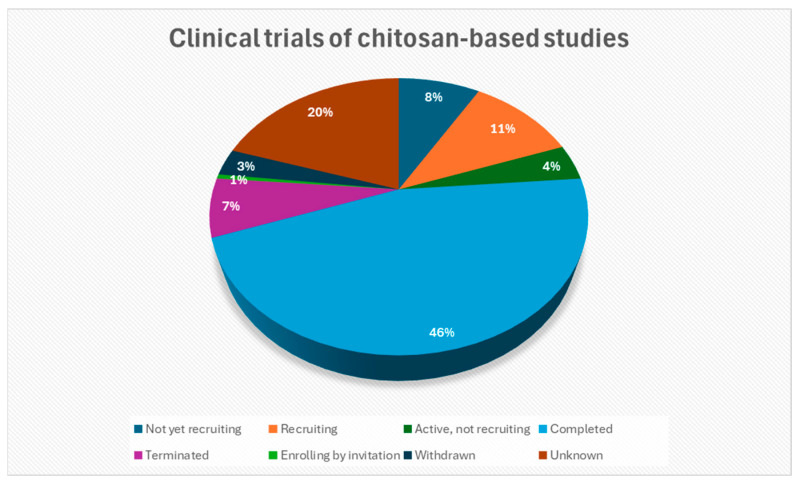
The recruitment statuses of clinical studies concerning chitosan-based materials (Search ClinicalTrials.gov for: Other terms: Chitosan|List Results|ClinicalTrials.gov, accessed on 14 September 2025) [171].

The translation of chitosan-based research into clinical practice necessitates the implementation of rigorous preclinical and clinical trials to guarantee the safety, efficacy, and regulatory conformance of the drug formulations. In this context, Wang et al. [167] conducted a registered clinical study (ClinicalTrials.gov NCT03907111) that compared chitosan fiber (CF) and chitosan sponge (CP) dressings in patients following abdominal surgery. The study found that CF dressings achieved quicker hemostasis and higher blood absorption than normal gauze. In a clinical experiment (ClinicalTrials.gov NCT03280849), Ramos-Zúñiga et al. [168] used a bilaminar chitosan scaffold to rebuild the sellar floor following pituitary surgery, resulting in watertight closure and high biocompatibility. No CSF fluid leaking, infection, or inflammation was noted throughout nearly three years of follow-up. Similarly, in a randomized trial of 59 cesarean-section patients, Kao et al. [169] (ClinicalTrials.gov NCT04211597) found that a chitosan-microencapsulated recombinant human epidermal growth factor (Me-EGF) spray plus silicone gel significantly improved wound healing and decreased Vancouver Scar Scale scores for vascularity, pigmentation, and pliability compared to silicone gel or no treatment. ElGendy et al. [170] conducted a double-blind RCT (NCT05212311) in 54 patients with mild–moderate cubital tunnel syndrome, a neurological illness, showing that adding chitosan nanoparticle gel phonophoresis to standard hand therapy improved ulnar nerve conduction, reduced pain, and enhanced hand function. The chitosan gel was applied through ultrasound (0.5 W/cm^2^, 3 MHz) three times per week for five weeks. There are currently 190 clinical studies involving the use of chitosan-based materials. Figure 8 indicates their recruitment statuses, while Table 3 summarizes selected chitosan-based clinical trials that have been completed, as indicated by their status of ‘Completed’ on ClinicalTrials.gov (Search ClinicalTrials.gov for: Other terms: Chitosan | List Results | ClinicalTrials.gov).

## 6. Conclusions, Future Work, and Recommendations

This review highlights two decades (2005–2025) of research on chitosan-based materials; these materials have shown remarkable promise for use in formulations. One of the key advantages of chitosan is its ease of chemical modification, which allows improvement of critical properties, e.g., solubility, which further expands its therapeutic potential. These materials’ flexibility to be used both as drug carriers and pharmacologically active agents drives their unique position in modern therapeutics. This has been evidenced from the in vivo and in vitro models, which support their anticancer, anti-inflammatory, antidiabetic, antihypertensive, and neuroprotective activities, while recent clinical trials highlight their safety, efficacy, and feasibility in diverse biomedical applications.

The significance of these findings lies in the uniqueness and ability of chitosan to bridge material science and pharmacology, offering a natural, modifiable, and safe platform for next-generation biomedical applications. Its dual role, such as therapeutic and structural, makes it a promising cornerstone for the design of multifunctional biomaterials.

Despite these advances, certain limitations remain; for instance, most studies do not adequately evaluate the cytotoxicity of chitosan-based materials, which is essential for ensuring their safety of use. Additionally, clinical translation remains limited, as some studies were withdrawn and others were terminated, as shown in Figure 8. Furthermore, a lot of findings are constrained in laboratory-scale studies.

Future research should focus on large-scale preclinical validation and well-designed clinical trials to confirm therapeutic efficacy and safety. Integration of computational modeling, green synthesis approaches, and advanced biofabrication (such as 3D printing and hybrid scaffolds) could accelerate the clinical translation of chitosan-based therapeutics, and lastly, the cytotoxicity of every chitosan-based material should be evaluated. With continued interdisciplinary collaboration, these materials hold strong potential to transform the treatment of chronic and degenerative diseases and could enter the market in high quantities in the near future.

## Figures and Tables

**Table 1:** Overview of Chemically Modified Chitosan Derivatives and their Biomedical Applications.

Type of Modification	Main Modification Site	Key Properties Improved	Biomedical Application	References
Quaternization of chitosan				
Quaternization of chitosan (mupirocin-loaded nanoparticles)	Amino group	Increased solubility, improved stability, enhanced drug encapsulation, sustained drug release	Wound healing, antibacterial delivery of mupirocin	[47]
Catechol grafting (dopamine modification, mussel-inspired)	Amino and hydroxyl group	Adhesiveness, self-healing ability, antioxidative activity, antibacterial properties	Wound healing and tissue engineering	[48]
Multi-component hydrogel network	Amino group	Improved water solubility, antibacterial activity, stronger electrostatic adhesion	Advanced wound dressings and tissue repair	[49]
Grafting of [2-(methacryloyloxy)ethyl] trimethyl ammonium chloride (MTAC) onto chitosan (CS-MTAC)	Amino group	Enhanced antibacterial activity, improved biocompatibility, better printability and rheological properties, sustained drug release (levofloxacin)	Wound healing	[50]
Genipin Crosslinked Quaternary Ammonium Chitosan Hydrogels	Amino group	Porous Structure, improved mechanical properties, adhesion Performance, Cytocompatibility:	Wound healing	[51]
Phosphorylation of chitosan				
Bioactive Glass/Phosphorylated Chitosan (BG/PCS) Composite Scaffold	Amino group	Enhanced compressive strength, improved apatite mineralization ability, stronger scaffold–cell interaction, better biocompatibility	Bone tissue engineering (scaffolds for bone regeneration)	[52]
Phosphorylated Chitosan (PCS) incorporated into Bioactive Glass (BG) scaffolds	Amino group	Enhanced compressive strength, improved apatite mineralization, increased hydrophilicity, better osteoblast adhesion and proliferation, strong scaffold–cell interaction, overall biocompatibility	Bone tissue engineering (bone regeneration scaffolds)	[53]
Phosphorylated Chitosan Hydrogel	Amino group	Enhanced osteogenic differentiation of osteoblasts, improved biocompatibility, stronger cell–material interaction	Bone tissue engineering, bone regeneration	[54]
Phosphorylated Chitosan scaffold	Amino group	Improved compressive strength, enhanced apatite mineralization, better hydrophilicity, increased osteoblast adhesion and proliferation, superior biocompatibility	Bone tissue engineering (scaffolds for bone regeneration)	[55]
Chitosan–Phosphorylated Chitosan Polyelectrolyte Complex (PEC) Hydrogel	Amino group	Water solubility of PCS, porous structure, excellent cytocompatibility, enhanced osteoblast adhesion, proliferation	Osteoblast carrier, bone tissue engineering scaffold	[56]
Sulfation of chitosan				
2-N,6-O Sulfated chitosan	Amino and hydroxyl group	Enhanced solubility, increased negative charge density, stronger binding to growth factors, promotion of periosteal stem cell recruitment and osteogenic differentiation	Bone regeneration, periosteum-guided tissue engineering	[57]
Regioselectively Sulfated Chitosan	Hydroxyl group	Increased negative charge density, improved anticoagulant activity, enhanced water solubility, stronger interactions with proteins and growth factors	Anticoagulant therapy, wound healing, tissue engineering, drug delivery	[58]
Sulfated Chitosan (SCS) Hydrogel	Hydroxyl group	Enhances angiogenesis, promotes growth factor binding, controlled release	Wound healing and tissue regeneration via angiogenesis promotion	[59]
2-N,6-O-sulfated chitosan	Amino and hydroxyl group	Robustly activates a moderate pro-inflammatory macrophage response, facilitates bone marrow stromal cell (BMSC) chemoattraction	Enhanced bone tissue development and bone regeneration, particularly in BMP-2-mediated osteogenesis and an ectopic ossification model	[60]
Sulfated chitosan	Hydroxyl group	Orchestrates macrophage	Angiogenesis-based diabetic wound repair	[61]
Carboxymethylation of chitosan				
Carboxymethyl Chitosan (CMCh) Hydrogel	Hydroxyl group	Enhanced solubility, high moisture retention, improved gel formation ability, antibacterial activity, macroporous 3D structure, good compressive strength, controlled drug encapsulation and sustained release	Drug delivery, wound dressing potential, broader tissue engineering uses	[62]
Carboxymethyl Chitosan Microgel	Hydroxyl group	Improved hydrophilicity and solubility, enhanced biocompatibility, strong gel-forming ability, controlled drug release, antibacterial activity	Drug delivery system	[63]
Carboxymethyl Chitosan Cryogel	Hydroxyl group	Enhanced hydrophilicity and swelling, strong binding affinity for metal ions, biocompatibility, ability to load and release antibiotics, antifouling properties	Drug delivery systems, potential biomedical scaffolds	[64]
Quaternized Carboxymethyl Chitosan (QCMCS)	Hydroxyl group	Rapid self-healing ability, injectability, pH responsiveness, strong mechanical strength, sustained drug release, biocompatibility, antibacterial activity	Drug delivery system	[65]
Carboxymethyl Chitosan (CMCS)/Polyvinylpyrrolidone (PVP)/Tannic Acid (TA) Hydrogel (CPT Hydrogel)	Hydroxyl group	pH-responsiveness, excellent self-healing and adhesion, antioxidant and antibacterial activity, hemostatic ability, swelling and porosity suited for wound exudate absorption, biocompatibility	Wound healing	[66]
Carboxymethyl Chitosan (CmCh)/Oxidized Alginate Self-Crosslinked Hydrogel	Hydroxyl group	Enhances solubility, biocompatibility, reactivity	Wound healing	[67]
Crosslinked Carboxymethyl Chitosan (CMCh)–Gelatin Scaffolds	Hydroxyl group	Enhanced swelling and moisture retention, improved mechanical properties, surface smoothness, antibacterial activity, cytocompatibility	Wound healing	[68]

**Table 2:** Chitosan-based materials in wound healing.

Type of Chitosan Modification/Product	Additional Components/Nanomaterials	Preparation Method	Key Properties	Antibacterial/Antioxidant/ Hemostatic Effects	In Vivo Outcomes	Animal Model	References
Sulfobetaine-modified chitosan cryogel (regioselective sulfation product)	ZIF-67, glucose oxidase (GOx), graphene oxide (GO)	Freeze-drying, in situ loading	Highly porous, fluid absorption, glucose-responsive behavior	Antibacterial, hemostasis	Healing promotion in diabetic full-thickness wounds	Mouse	[91]
Carboxymethyl chitosan/oxidized alginate injectable hydrogel (CMC-based product)	-	Self-crosslinking (Schiff base)	Injectable, self-healing, tunable mechanics	Antibacterial	Accelerated wound closure in infected wound model	Rat	[67]
Quaternized chitosan-based hydrogel (QCS-MA/PVA/ZIF-8/NMN)	ZIF-8 nanoparticles, nicotinamide mononucleotide (NMN)	UV photo-crosslinking	Improved mechanical strength, adhesion, sustained NMN/Zn^2+^ release	Antibacterial	Accelerated wound closure in infected wound model	Rat	[92]
Chitosan/PVA composite freeze-dried sponge	Oyster shell powder (CaCO_3_)	Freeze-drying porous sponge	Porous structure, Ca^2+^ release, high absorption	Antibacterial	Hemostasis models (rat liver and tail): reduced blood loss and time	Rabbit	[93]
Triple-responsive chitosan	-	Quercetin-Cu	Improved mechanical strength	Antioxidant, hemostasis	Diabetic wound healing: enhanced fibroblast remodeling and angiogenesis; PI3K-AKT activation	Rat	[94]
Thermosensitive injectable self-assembled hydrogel (TISH(GR))	Granulocyte-macrophage colony-stimulating factor (GM-CSF), Resveratrol	Thermosensitive sol–gel transition	Porous structure, injectable, sustained drug release, immunomodulatory	-	Rat model (Wistar rats): High-fat diet-induced periodontitis.	Rat	[95]
Quaternized chitosan-based hydrogel	-	One-pot method	Adhesion, porous structure, injectable	Antibacterial, hemostasis	Significantly enhances collagen deposition, reduces senescent cell accumulation, and accelerates wound closure	Rat	[96]
Chitosan-MXene-silver nanocomposite film	Silver nanocomposite	-	Mechanical strength	Antioxidant, antibacterial, hemostasis	Wound closure, tissue regeneration	Rat	[97]
Chitosan–gelatin–PEG hydrogel	*Aloe vera* and *Curcuma longa* extracts encapsulated in alginate-CaCl_2_	Freeze–thaw, freeze-drying	Porous, mechanical strength	Antioxidant, antibacterial	Wound closure, fibroblast proliferation	Rat	[98]
Chitosan-sericin cryogel	Sericin, ε-polylysine (EPL)	Crosslinking; freeze-drying	Porous, mechanical strength	Antibacterial, hemostasis	Wound closure	Rat	[99]
Oriented periostracum cicadae chitosan hydrogel (O-CH@Ag)	AgNPs	-	Mechanical strength	Antibacterial, hemostasis	Wound closure	Rat	[100]
Niaouli oil-loaded chitosan hydrogel (CS-N)	Niaouli essential oil	-	Mechanical strength	Antioxidant, antibacterial	Wound closure	Rat	[101]
Xylan–Chitosan biopolymeric films	Deep eutectic solvent (DES)	Solvent-casting films	Mechanical strength	Antibacterial	Wound closure	-	[102]
Gelatin/quaternized chitosan-based macroporous sponge (GQ2O)	Procyanidin (polyphenol), soybean lecithin (blowing agent), genipin	Mechanical stirring, freeze-drying	Mechanical strength	Antioxidant, antibacterial, hemostasis	Wound closure	Rat	[103]

**Table 3:** Some of the clinical trials involving the use of chitosan-based materials (Search ClinicalTrials.gov for: Other terms: Chitosan | List Results | ClinicalTrials.gov, accessed on 14 September 2025) [171].

Study Title	NCT Number	Conditions	Interventions	Sponsor
Use of Chitosan Powder in Loop Electrosurgical Excision Procedure	NCT05661708	Vaginal Bleeding Loop Electrosurgical Excision	Drug: Chitosan	Erzincan Military Hospital
Clinical Assessment of Chitosan Nanoparticles on Periodontal Problems Post Steroidal Inhalation in Asthmatic Patients: Phase I Trial	NCT06525363	Periodontal Diseases	Drug: Chitosan	Deraya University
A Crossover Trial of Chitosan Oligosaccharide on Post Prandial Glucose Control in Subjects With Normal, IFG and IGT	NCT03650023	Impaired Fasting Glucose Impaired Glucose Tolerance	Dietary Supplement: Chitosan Oligosaccharide (GO2KA1)	Yonsei University
Clinical Investigation of Bleeding Reduction Efficacy on Toothpaste Containing 1.05% Chitosan	NCT06955871	Bleeding Gum Gingivitis	Other: Toothpaste containing 1.05% Chitosan	Colgate Palmolive
Efficacy and Safety of HEP-40 Chitosan for Mild to Moderately Elevated Cholesterol	NCT00454831	Hypercholesterolemia	Device: HEP-40 chitosan	DNP Canada
Efficacy and Safety of a Fungal Chitosan on the Body Weight Reduction in Overweight and Obesity Volunteers	NCT02246699	Overweight and Obese Volunteers.	Device: KiOnutrime^®^-Cs Other: Placebo	Kitozyme
Compare the Hemostatic Effectiveness of Chitosan Gauze With Traditional Gauze on Open Wound on 10 Participants.	NCT03907111	Hemostasis	Device: Chitosan Gauze Device: placebo	Tri-Service General Hospital
Efficacy of Ozone Gel, Doxycycline Saturated Chitosan Dressing Versus Alveogyl	NCT05875506	Osteitis Dry Socket	Drug: doxycycline hyclate saturated chitosan dressing	Minia University
Efficacy of the Combination of Isosorbide Dinitrate Spray and Chitosan in Diabetic Foot Ulcers	NCT02789033	Diabetic Foot Ulcers	Drug: Chitosan Drug: Placebo	University of Guadalajara
Clinical and Radiographic Outcomes of PRF, Chitosan, and Blood Clot in Regenerative Endodontics of Molars	NCT07119619	Regenerative Endodontics	Procedure: Regenerative Endodontic Treatment Using a Chitosan as a Scaffold Procedure: Regenerative Endodontic Treatment Using a Platelet Rich Fibrin as a Scaffold	Inonu University
Evaluation of Chitosan Scaffold and Mineral Trioxide Aggregate Pulpotomy in Mature Permanent Molars With Irreversible Pulpitis	NCT04308863	Pulpitis—Irreversible	Drug: Chitosan scaffold/MTA pulp dressing material	Nourhan M.Aly
MicroFIBERgut: Effects of Lifestyle Changes and Chitosan on Gut Microbiota and Weight Management	NCT04551365	Obesity Lifestyle	Dietary Supplement: Chitosan Other: Placebo	University of Iceland
A Randomized Trial to Assess the Efficacy and Safety of GO2KA1(Chitosan Oligosaccharide)on Blood Glucose Control	NCT01496820	Impaired Fasting Glucose, Newly diagnosed Type 2 Diabetes	Dietary Supplement: GO2KA1 Dietary Supplement: Placebo	Yonsei University
A Study to Evaluate Chitosan Chewing Gum in Patients With Chronic Kidney Disease	NCT01475760	Chronic Kidney Disease	Other: K2CG chewing gum (20 mg chitosan) Other: K2CG chewing gum (60 mg chitosan)	Denver Nephrologists, P.C.
Comparison of Chitosan, Ankaferd and Tranexamic Acid in Dental Extraction in Liver Pre-Transplant Children	NCT06457360	Liver Cirrhosis	Drug: tranexamic acid Drug: Ankaferd Blood Stopper Drug: Chitosan	British University In Egypt
Slow-release Tb4 Collagen and Chitosan Porous Sponge Scaffolds Skin Substitute Treatment is Difficult to Heal Wounds	NCT02668055	Wounds	Biological: TB4	Chinese PLA General Hospital
Beta-glucan-chitin-chitosan Polymer Supplement in Overweight/Obese Subjects: Cardiovascular Risk Biomarkers (QUITOVASC)	NCT06622447	Dietary Exposure	Dietary Supplement: βGluCnCs intervention Dietary Supplement: Placebo intervention	Fundació Institut de Recerca de l’Hospital de la Santa Creu i Sant Pau
Phosphate Binding of Chitosan Chewing Gum in Patients With Chronic Kidney Disease (CKD)	NCT01341691	Chronic Kidney Disease	Other: chewing gum	Denver Nephrologists, P.C.

## Data Availability

No new data were created or analyzed in this study. Data sharing is not applicable to this article.
